# Perspective: Structure determination of protein-ligand complexes at room temperature using X-ray diffraction approaches

**DOI:** 10.3389/fmolb.2023.1113762

**Published:** 2023-01-23

**Authors:** Michael A. Hough, Filippo Prischi, Jonathan A. R. Worrall

**Affiliations:** ^1^ School of Life Sciences, University of Essex, Colchester, United Kingdom; ^2^ Diamond Light Source Ltd., Harwell Science and Innovation Campus, Didcot, United Kingdom

**Keywords:** X-ray crystallography, time-resolved, ambient temperature, spectroscopy, protein-ligand complexes

## Abstract

The interaction between macromolecular proteins and small molecule ligands is an essential component of cellular function. Such ligands may include enzyme substrates, molecules involved in cellular signalling or pharmaceutical drugs. Together with biophysical techniques used to assess the thermodynamic and kinetic properties of ligand binding to proteins, methodology to determine high-resolution structures that enable atomic level interactions between protein and ligand(s) to be directly visualised is required. Whilst such structural approaches are well established with high throughput X-ray crystallography routinely used in the pharmaceutical sector, they provide only a static view of the complex. Recent advances in X-ray structural biology methods offer several new possibilities that can examine protein-ligand complexes at ambient temperature rather than under cryogenic conditions, enable transient binding sites and interactions to be characterised using time-resolved approaches and combine spectroscopic measurements from the same crystal that the structures themselves are determined. This Perspective reviews several recent developments in these areas and discusses new possibilities for applications of these advanced methodologies to transform our understanding of protein-ligand interactions.

## Introduction

The interaction between a protein and a small molecule ligand is a fundamental aspect of many biochemical processes ranging from molecular oxygen (O_2_) binding to signal transduction and gene expression. Often the life-time of the protein-ligand complex is tuned to dictate its function. In this respect, protein ligand interactions can display life-times ranging from microseconds to days. Complex life-times are determined by the ligand off-rate, with a high off-rate commonly associated with transient complexes and resulting in a dissociation equilibrium constant (*K*_D_) in the milli to micromolar range, whereas static complexes have a significantly lower off-rate and subsequently *K*_D_s into the nanomolar range and beyond. Obtaining high-resolution structural information for protein-ligand complexes provides direct information on the mode of interaction and is particularly powerful when combined with suitable biophysical methods, such as calorimetry or surface plasmon resonance that enable thermodynamic and kinetic parameters of the binding interaction to be quantified. Tremendous effort has been invested into developing methods to obtain structures of protein-ligand complexes, with orders of magnitude improvements in sample throughput and data analysis being achieved, particularly for the application of early fragment-based drug discovery (Collins et al., 2017; Krojer et al., 2017; Pearce et al., 2017; Thomas et al., 2019).

Despite the notable successes, challenges remain. For example, it is recognised that data collection under the cryogenic conditions (100 K) that are standard in X-ray crystallography may result in binding modes or interactions that are not fully consistent with those occurring under physiological conditions (Helliwell, 2020). A fascinating example was recently provided by a systematic comparison of the binding of fragments to the enzyme protein tyrosine phosphatase, PTP1B (Mehlman et al., 2022). Here, fragments were soaked into crystals that were then either cryo-cooled conventionally, harvested for room temperature data collection in loops or measured *in situ* within crystallisation plates. Intriguingly, significant differences were observed for some fragments between the 100 K structures and those at room temperature despite the original soaking and presumably binding being identical (Mehlman et al., 2022). In this study, different temperatures revealed distinct binding poses and protein-ligand interactions, novel binding sites and changes in water structure together with a previously uncharacterised allosteric binding site.

Additionally, the X-rays used to determine structures can themselves alter the structure of the crystalline protein through radiation damage (Henderson, 1995; Owen et al., 2006), which is a pressing concern for enzymes containing redox active metal centres such as iron, which are susceptible to radiation induced oxidation state changes (Henderson, 1995; Owen et al., 2006; Pfanzagl et al., 2020; Hough and Owen, 2021; Lučić et al., 2021; Worrall and Hough, 2022). Furthermore, it can be challenging to access and structurally characterise transient protein-ligand complexes, and also transient species such as catalytic intermediates using conventional X-ray crystallography. These challenges have been a major driver in the development of time-resolved crystallography using synchrotron and X-ray free electron laser (XFEL) facilities.

In this Perspective, we review recent methodological advances relevant to structure determination of validated, high-resolution protein-ligand complexes and provide several examples of how these approaches have the potential to elevate our understanding of ligand binding.

## X-Ray structure determination at room temperature

Early X-ray crystal structures were almost exclusively obtained at room temperature with crystals typically mounted in capillaries. Radiation damage concerns and increasingly brilliant X-ray synchrotron beamlines led to the development of highly effective cryo cooling methods with almost all structures subsequently determined at 100 K. Such cryogenic structures have led to tremendous insights into protein-ligand binding over many decades. However, the question of physiological relevance on a crystal complex determined at 100 K arises (Helliwell, 2020), and thus ligand binding may well be affected by cryo cooling leading to the masking of alternate conformational states (Fraser et al., 2011). Fischer et al. (2015) demonstrated that on shifting the temperature of a crystal the population of conformational states can be perturbed towards energetically less-accessible states and result in the detection of new binding sites for low-affinity (transient) or less-soluble ligands. A more recent study highlighted the structural plasticity of the active site cavity of SARS-CoV-2 3CL Mpro in room temperature structures and that such non-cryogenic structures provided a better starting point for computational or experimental ligand binding studies than did the 100 K equivalent (Kneller et al., 2020; Ebrahim et al., 2021). Moreover, multi-temperature, multi-crystal studies using PTP1B revealed low occupancy conformational states and consequently allosteric binding hotspots that were not apparent in the conventional 100 K structure (Keedy et al., 2018).

A simple approach to determine room temperature crystal structures is to measure data directly from crystals within suitable crystallisation plates. The first dedicated beamline to do this was the versatile macromolecular crystallography *in situ* (VMXi) beamline at Diamond Light Source (Sanchez-Weatherby et al., 2019), with several additional instruments now in operation worldwide (Okumura et al., 2022). Alternative approaches involve measuring data from crystals between sheets of low background material such as mylar (Broecker et al., 2018). Usually in these approaches data are merged from a small number of protein crystals to produce a complete dataset, with this approach having tremendous potential for room temperature fragment screening for protein ligand interaction characterisation.

Fragment screening is now a primary method at the beginning of the drug development pipeline and relies on the ability to accurately identify the binding modes of small fragment ligands to identify drug sites and allow for fragment growing/joining into drug like candidate compounds (Thomas et al., 2019). X-ray crystallography offers a unique opportunity to obtain this information, as standard biophysical approaches often fail to identify binding fragments, as their *K*_D_ are usually in the millimolar range. Room temperature fragment screening approaches offer advantages over conventional cryo cooled approaches for reasons outlined above. Thus, development of new methods to determine structures of protein-ligand complexes with high throughput at room temperature in a manner that is efficient both in crystals and for often expensive ligands is desirable. A recent example in this area is a microfluidic protein crystal array device that has been developed to efficiently mix multiple ligand compounds with groups of crystals and allow for X-ray data collection within the device (Maeki et al., 2020). This approach was applied to determine several different structures of complexes between thaumatin, lysozyme and trypsin and several different ligands with between 3 and 20 protein crystals being used to determine each complex structure. Notably, the room temperature trypsin-ligand complexes revealed differences in ligand coordination compared to cryogenic structures, further illustrating advantages for downstream development of initial fragment hits (Maeki et al., 2020). Specifically, the ligands 5-chlorotryptamine and 5-methoxytryptamine displayed alternative conformations only at 100 K, suggested by the authors to be possibly the result of a cryo artefact such as the presence of cryoprotection agents. Similarly, the ligands benzamidine and aniline exhibited additional binding sites at 100 K compared to room temperature, which were some distance from the trypsin binding site and thus assigned to be physiologically non-relevant.

## Serial crystallography and time-resolved approaches

A transformative development in structural biology has been the emergence of serial crystallography, where large numbers (> 1000) of microcrystals (typically 10 µm or smaller maximum dimension) are used for X-ray diffraction in sequence to assemble a complete dataset (Chapman et al., 2011; Chapman et al., 2014). The crucial advantage of this method is that each crystal is exposed either for tens of femtoseconds when using an XFEL or tens of milliseconds when using a synchrotron radiation source (Weinert et al., 2017). The short exposures in turn leads to structures where the electron density maps and related structures are essentially free of manifestations of X-ray induced chemistries and also to the exciting possibility of determining time-resolved structures with a defined time delay between reaction initiation and structure determination (Orville, 2020; Malla and Schmidt, 2022).

**FIGURE 1:**
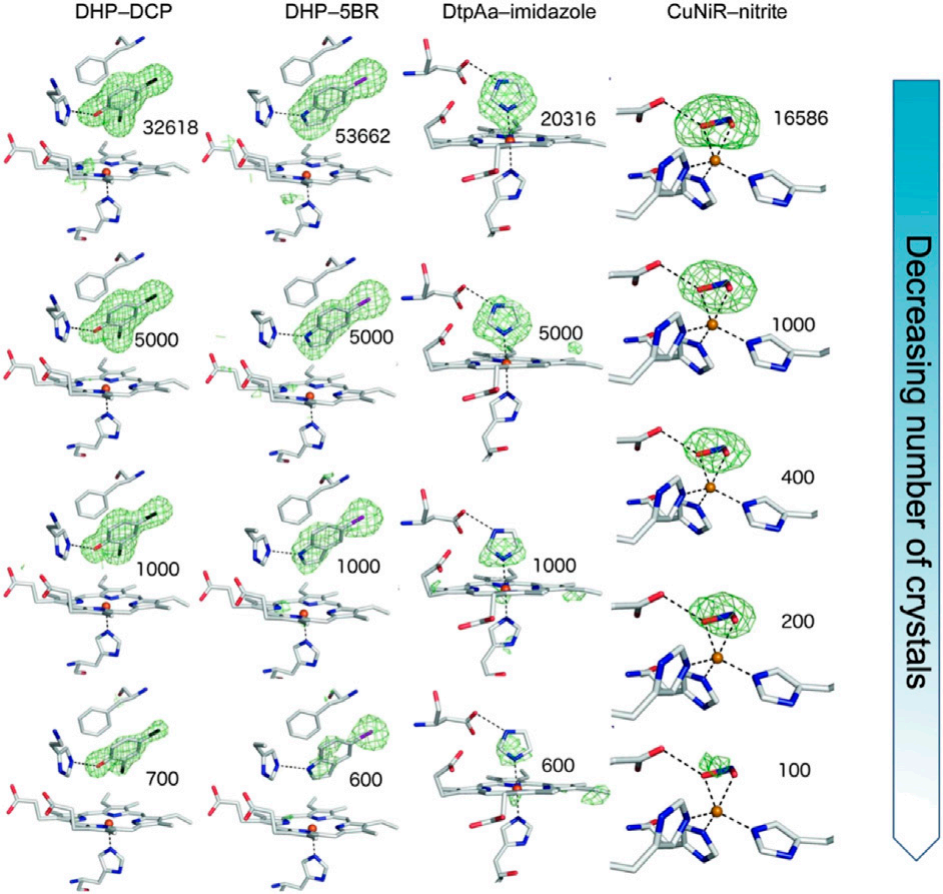
SFX results showing Fo-Fc simulating annealing OMIT electron density contoured at 3 *σ* (green) for different sizes of ligands in the active site pocket (depicted in sticks) of the enzymes dehaloperoxidase B (DHP) from *Amphitrite ornata*, dye-type decolorizing peroxidase Aa (DtpAa) from *Streptomyces lividans* and copper nitrite reductase (CuNiR) from *Achromobacter cycolclastes*. The ligands 2, 4 dichlorophenol (DCP), 5-bromoindole (5BR), imidazole and nitrite, respectively were soaked into the microcrystals. The top row represents the full datasets obtained with subsequent rows being from subsets of the data of decreasing size, numbers shown indicating the number of diffraction patterns comprising each dataset. Remarkably, data from only a few hundred to a few thousand microcrystals reproduced the electron density for the ligand enabling accurate modelling. This result opens the door to high throughput fragment screening/structure-based drug design using SFX. Figure adapted from (Moreno-Chicano et al., 2019).

We have shown the utility of serial femtosecond crystallography (SFX) at the SACLA XFEL in Japan to determine high-quality structures of protein-ligand complexes of iron and copper-containing proteins (Moreno-Chicano et al., 2019). Ligands smaller (< 200 Da) than those generally used in fragment-based drug design (≥ 300 Da) were investigated with their presence, occupancy and orientation clearly identified from the SFX data which was obtained using fixed target silicon chips (Owen et al., 2017) that were loaded with ligand soaked microcrystal slurries of the respective protein. The number of crystals required to identify ligand binding modes unambiguously was explored, surprisingly revealing that comparatively small datasets derived from < 1,000 crystals were sufficient to identify the ligand, Figure 1. Thus this approach, and others (Nass Kovacs, 2021), has potential for pursuing challenging targets (e.g., low crystal or ligand quantities) in structure-based drug design using room temperature SFX.

Using SFX to obtain structures of enzyme-substrate or enzyme-product complexes provides the essential starting point to determine time-resolved structures of transient states or enzyme intermediates. Several different methods have been developed to initiate reactivity within microcrystals. The most intuitive approach is to rapidly mix microcrystals with a suitable reagent solution at a defined point prior to X-ray exposure, in a manner analogous to stopped-flow enzyme kinetics. While conceptually simple, there are many technical challenges related to mixing rates and optimal homogeneous reaction initiation. Simple mixing of a protein and ligand can be achieved using a mix-and-inject system (Olmos et al., 2018; Pandey et al., 2021). Alternatively, droplets containing ligand solutions may be acoustically ‘fired’ at microcrystals that are either within droplets (Hejazian et al., 2020) on a tape drive (Butryn et al., 2021) or contained within a silicon fixed target (Mehrabi et al., 2019a). Whichever approach is used, the timescales accessible are limited by diffusion of the ligand into the crystal which is typically in the millisecond range.

Another approach for reaction initiation or ligand binding in a protein crystal is the use of light. Initially this was applied to naturally photosensitive systems such as rhodopsins (Panneels et al., 2015) or fluorescent proteins (Coquelle et al., 2018) and more recently to photoenzymes (Sorigué et al., 2021). In order to expand the applicability of light activation, significant progress has been made in the development of different photocage systems where a ligand molecule is released upon uncaging *via* a pulse of light of the appropriate wavelength, typically delivered using a laser pulse (Monteiro et al., 2021). This approach has the potential for much faster reaction initiation as diffusion into the crystal is not a significant factor, due to pre-soaking with the photocage. A recent example has been the use of a nitric oxide (NO) photocage (Namiki et al., 1997) to initiate NO ligand binding in cytochrome P450 nitric oxide reductase (Tosha et al., 2017; Nomura et al., 2021). Using time-resolved SFX a transient NO bound heme intermediate was identified which formed after a 20 ms timed delay following photolysis of the caged NO (Nomura et al., 2021). A further example of the application of photocages to release a ligand substrate has been reported for the enzyme fluroacetate dehalogenase (Mehrabi et al., 2019b). Here, uncaging releases the ligand substrate fluroacetate which was followed by time-resolved serial synchrotron crystallography to reveal coupled allosteric motions between the two active sites of the dimeric enzyme (Mehrabi et al., 2019b).

## Combining spectroscopic and structural approaches to define protein-ligand complexes

A major challenge in structural approaches to study protein-ligand complexes is the assignment of structures to the appropriate state or complex. In many cases the electron density may be ambiguous and can represent a mixture of various occupancies and intermediates within crystals. A further confounding factor is that the X-rays themselves can change the structure, particularly for proteins containing redox centres such as transition metals or flavins. A tremendously useful tool to help assign structures to redox states of enzymes is to combine crystal spectroscopies with X-ray diffraction. Most commonly used has been visible spectroscopy which is very powerful when a chromophore is present, for example in heme proteins (von Stetten et al., 2015; Kekilli et al., 2017; Lučić et al., 2021). Other methodology has involved X-ray absorption spectroscopy from single crystals, resonance and non-resonance Raman, infrared and fluorescence spectroscopies (Kekilli et al., 2014; von Stetten et al., 2015). Typically, these approaches allow the electronic state of the redox metal ion, which can be ligand sensitive, to be explicitly identified and also allow the effects of radiation damage from the X-rays to be monitored and with sufficient care, at least partly ameliorated.

**FIGURE 2:**
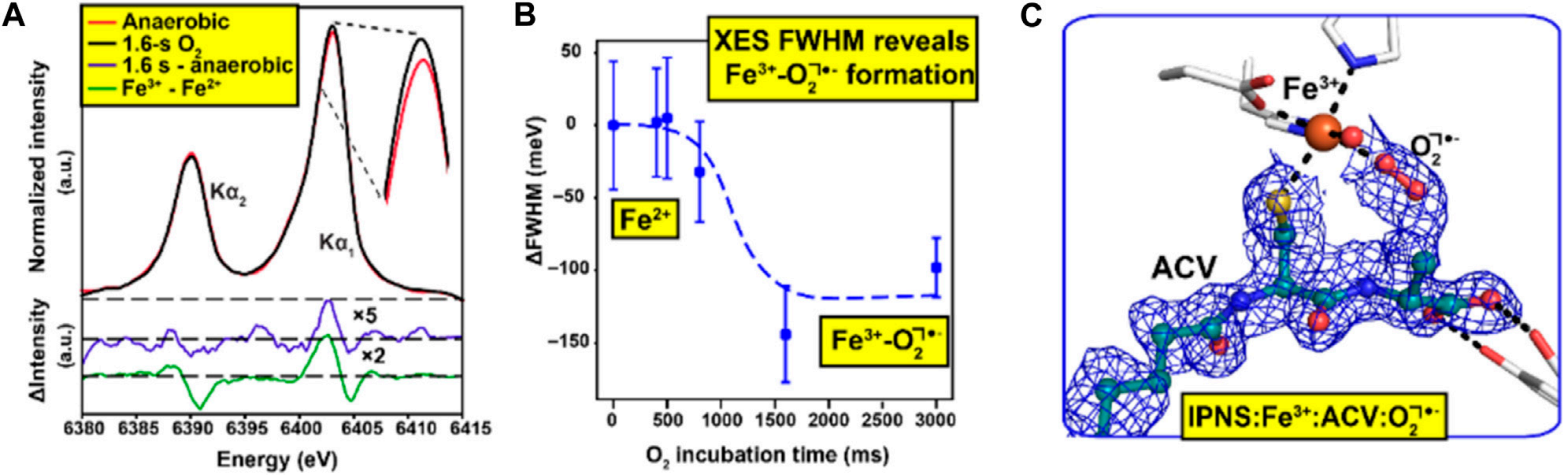
Combination of spectroscopic and XFEL data to understand ligand binding [*d*-(L-α-aminoadipoyl)-L-cysteinyl-D-valine (ACV) and O_2_] to the enzyme isopenicillin *N*-synthase (Rabe et al., 2021). Initial XFEL data were obtained for the enzyme with the substrate ACV, a precursor to for all natural cephalosporins and penicillins. Reactivity within microcrystals was initiated by passing them through an O_2_ chamber and SFX data were measured in a time-dependent manner. X-ray emission spectroscopy (XES) data **(A)** was obtained using the same X-ray pulse that generated the diffraction patterns allowing a two-step transition between the resting states Fe(II) and intermediate Fe(III)-O_2_^•−^states to be characterised over a wide range of reaction times **(B)** SFX data were obtained at a time point of 1,600 ms such that the Fe(III)-O_2_^•−^intermediate had fully formed **(C)** Figure adapted from (Rabe et al., 2021).

It is particularly important but also challenging to have spectroscopic validation for time-resolved ligand binding crystallographic experiments. In experiments using XFELs with sufficiently short pulse length, the crystal is destroyed by the extremely intense X-ray pulse, but diffracted X-rays leave the crystal sufficiently quickly that in many cases no evidence of radiation damage is apparent. The key challenge here for spectroscopy is to ensure that the spectroscopic data reflect the same crystal volume and timescale as the structural data so that the two are properly correlated (Srinivas et al., 2020). A key development to achieve this has been measurement of X-ray emission spectroscopy (XES) data using the same very short (femtoseconds) X-ray pulse that is used for X-ray diffraction data collection. This has been demonstrated using a von Hamos spectrometer system to record emission spectroscopy data from the same crystals and X-ray pulse at the LCLS XFEL (Stanford, United States) using microcrystals within droplets on a tape drive system (Butryn et al., 2021). Here, XES has been applied to identify different ligand states of the diiron active site in methane monooxygenase, after O_2_ binding (Srinivas et al., 2020) and the non-heme iron isopenicillin *N*-synthase bound to its substrate *via* a Cys ligand and activated by the binding of the co-substrate O_2_ (Figure 2) (Rabe et al., 2021) at different time points.

## Discussion

New X-ray methodologies have enabled researchers to go beyond the classic paradigms of a single structure of a protein-ligand complex at 100 K, but as yet, the number of crystal structures deposited in the Protein Data Bank using these new methods is relatively limited in part due to the novelty but also due to scarcity of instrumentation and expertise in some cases. For example, XFEL access is very limited with only five facilities operational worldwide, with all but two of these having only come online in the last 5 years. Similarly, few beamlines at synchrotron sources are equipped for serial crystallography or coupled to spectroscopy readout from crystals, and importantly, expertise in these approaches is unevenly spread within the structural biology community. As instrumentation develops and becomes more widespread, and more research groups become involved we anticipate a major increase in the number of room temperature and/or time-resolved structure determinations for protein-ligand complexes.

As well as the data generated from the combined X-ray and spectroscopic approaches, it is important to point out that for a full understanding of complex enzyme mechanisms and protein-ligand binding events it is necessary to combine computational approaches. In X-ray diffraction data, it is typically not possible to identify protonation states of amino acid residues or ligands. However, this information is critical to understand mechanism of action of ligand binding. For example, quantum mechanical/molecular mechanical (QM/MM) simulations of copper nitrite reductase clarified the protonation states of a catalytically important aspartate residue together with that of the substrate, intermediates and products (Sen et al., 2021). However, the combination of molecular simulation with experimental structure determination with each informing the other remains in its infancy, but as computational power enables more rapid simulations, we anticipate an increase in the number of combined studies in the coming years. Such an approach will be particularly important for the study of enzyme reactions where simulations allow access to transient ligand states that cannot be experimentally captured even by time-resolved methods and allow reaction coordinates to be produced linking together experiments and structures of different intermediate states. Almost by definition, time-resolved structures are obtained at ambient temperature and such high-quality structures close to physiological temperature provide the best starting model for computational analysis.

Context for the above is provided by further development of ultrahigh throughput structure determination of protein-ligand complexes at 100 K. This field has been pioneered by the XCHEM platform at Diamond Light Source (Douangamath et al., 2021) with a far higher throughput facility planned in the future. Extensive computational software development has been undertaken to assess low occupancy binding and to enable fragment binding examination, fragment joining and expansion (Pearce et al., 2017). These tools will be highly applicable to work at ambient temperature and we envisage combined studies using both crystals under cryogenic conditions and at ambient temperature (Krojer et al., 2020). While several studies report ligand binding at room temperature and 100 K (Maeki et al., 2020; Mehlman et al., 2022), a full understanding of which protein-ligand interactions are likely to display such differences remains elusive. Similarly benefiting from computational power are docking software and machine learning (ML) algorithms for ligands and drug molecules identification and/or generation. Several deep neural networks approaches have been shown to rapidly generate sets of high-affinity binders by, for example, designing ligand shapes complementary to protein binding pockets (Skalic et al., 2019). Despite these advances, explicitly accounting for protein flexibility and, more importantly, ligand-induced protein conformational changes in ML approaches is still a challenge. On the contrary, ensemble docking approaches have shown great potential to overcome this limitation by docking ligands against a set of different conformations of the protein obtained from molecular dynamics simulations (Korb et al., 2012). However, proper selection of conformers is crucial for the success of the docking calculations. Recent methods to enrich ensembles in ligand-bound-like conformations using a multistep strategy for clustering binding site shapes consistently reproduced the correct conformation (∼1 Å RMSD from experimental ligand-bound structure) for all proteins tested (Basciu et al., 2019). As these methods develop, candidate molecules for experimental studies of protein-ligand complexes will become available more quickly requiring higher throughput experimental methods to keep pace.

In conclusion, recently developed methods tremendously enhance our ability to understand the interactions between proteins and their ligands. Synergies between these methods and envisaged future developments suggest a bright future for time-resolved, ambient conditions structural biology of protein-ligand complexes.

## Data Availability

The original contributions presented in the study are included in the article/supplementary materials, further inquiries can be directed to the corresponding authors.
